# Experimental Research Models to Assess the Cross-Reactivity between Can f 5 and Human PSA—Two Different Perspectives

**DOI:** 10.3390/ijms231911223

**Published:** 2022-09-23

**Authors:** Kinga Lis, Natalia Ukleja-Sokołowska, Rafał Adamczak, Zbigniew Bartuzi

**Affiliations:** 1 Department of Allergology, Clinical Immunology and Internal Medicine, Ludwik Rydygier Collegium Medicum in Bydgoszcz, Nicolaus Copernicus University in Toruń, ul. Ujejskiego 75, 85-168 Bydgoszcz, Poland; 2 Department of Obstetrics and Gynecology, Ludwik Rydygier Collegium Medicum in Bydgoszcz, Nicolaus Copernicus University in Toruń, ul. Ujejskiego 75, 85-168 Bydgoszcz, Poland

**Keywords:** allergy, cross-reactive allergens, inhibition tests, Can f 5, PSA

## Abstract

The similarity in antigenic structures, including the degree of correspondence between the amino acid sequence and the spatial structure, is not always reflected in the actual cross-reactivity of allergens. Inhibition tests appear to be an invaluable tool for assessing potential cross-reactivity between allergens under natural conditions. In this publication, we present two experimental models of inhibition tests: solid phase (SP-IT) and liquid phase (LP-IT). As an exemplary research model, we used the cross-reactivity between human PSA and anti-Can f 5 IgE antibodies. We performed an SP-IT model using a microplate coated with human PSA. The LP-IT model was based on mixing anti-Can f 5 IgE positive serum with a material containing human PSA. Anti-Can f 5 IgE and PSA concentrations before and after inhibition were measured to evaluate inhibition effectiveness. The results of the performed experiments showed that both proposed models of inhibition tests are an effective tools for studying cross-reactive relationships between antigens. In the SP-IT, the concentration of anti-Can f 5 decreased by 21.6% and, in the LP-IT, it decreased by 34.51%. In turn, the PSA concentration in the SP-IT decreased by 11.25% and, in the LP-IT, it decreased by 15.49%. In conclusion, both the SP-IT and the LP-IT seem to be effective tools for assessing the actual cross-reactivity between different allergens.

## 1. Introduction

Allergic cross-reactivity occurs when the body’s immune system identifies the proteins from different sources as being similar. Antibodies specific to one of these proteins may also recognize similar epitopes present in other proteins. In this situation, when an individual comes into contact with either of these proteins, the immune system can react in the same way, which in some cases can cause allergic symptoms. The immune system reacts in this way because these proteins are similar in structure and may have similarly structured epitopes that are recognized by antibodies specific for either of these proteins. The possibility of cross-reactivity can cause difficult diagnostics but also can lead to various unforeseen and unexpected allergic reactions in allergic individuals [1]. This phenomenon is clinically significant because, in the case of cross-reactivity, specific clinical signs of training unexpectedly develop in a person exposed to a particular allergen without prior exposure to it [2].

The clinical manifestation of cross-reactivity is not always proportional to the actual structural similarity of the proteins. It is also not always associated with the phylogenetic relationship of allergen sources. In some cases, we do not observe clinical manifestations proportional to the significant structural similarity of allergens. On the other hand, it is possible that, despite the slight antigenic similarity of allergens from different sources, the clinical manifestation of cross-reactivity after exposure to these allergens is significant [1,3,4]. Currently, there are no standardized methods for testing cross-reactivity between allergens.

In the following research, two methods, based on the determination of the amino acid sequence as well as various techniques of spatial imaging of the protein structure, are proposed. These methods intend to help in estimating the likelihood of an allergic cross-reaction [4,5].

*Canis familiaris* prostatic kallikrein (Can f 5) is also known as arginine esterase. This is a 28 kDa protein found in the urine and fur of a dog. It is secreted in the prostate gland under the influence of androgens such as the prostate-specific antigen (PSA) in humans. Can f 5 is not found in female dogs. Its concentration is lower in young animals, and the castration of the animal causes a practically complete reduction in the secretion of this allergen [6].

Prostate-specific antigen (PSA), a 33–34 kDa glycoprotein, is a serine protease member of the human kallikrein family. It is produced in both prostate tissue and secreted into seminal fluid. It is one of the proteolytic enzymes in male semen. Its physiologic function is to liquefy semen from its gel form [7,8]. It was found that PSA carries high homology to the canine prostatic kallikrein, which was identified as Can f 5. For this reason, male sperm PSA may likely cause an allergic reaction after unprotected sexual contact in women allergic to canine kallikrein (Can f 5) [9]. Few case reports of this type have been published [10,11,12,13]. In some of them, additional studies were carried out and showed that the causative agent may be the ability of the anti-Can f 5 IgE antibody to recognize PSA as a target antigen [14,15]. There are also assumptions that allergy to Can f 5 in women may result in reproductive failure and should be considered as one of the immunological causes of infertility [16].

In our earlier publication [17], we demonstrated the ability of anti-Can f 5 IgE antibodies to partially bind human PSA. This property is likely due to the similarity between the canine kallikrein molecule and human prostatic kallikrein, which results in the cross-reactivity of antibodies specific to these proteins [18]. In the present work, we have attempted to confirm this dependence by using extensive, multidirectional experimental models. This approach allowed us to study the relationship between Can f 5 and human PSA and the cross-reactivity of antibodies specific to these antigens from several perspectives.

The aim of the study was to evaluate the usefulness of various models of inhibition tests in studies on cross-reactivity of allergens. We proposed two independent research models based on the inhibition assays we described before. The ability of anti-Can f 5 IgE antibodies to block human PSA had been the subject of our research previously [17].

## 2. Results

**Figure 1:**
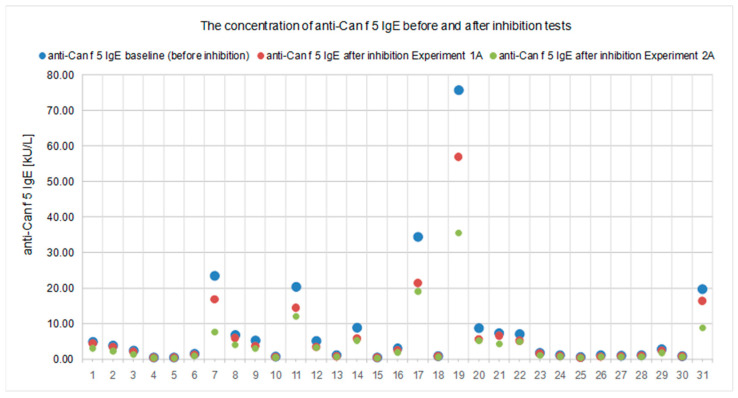
Anti-Can f 5 IgE concentration before and after inhibition tests with human PSA, in each case, in the experimental models used. The inhibition was statistically significant in each of the proposed procedures (*p* < 0.05).

**Figure 2:**
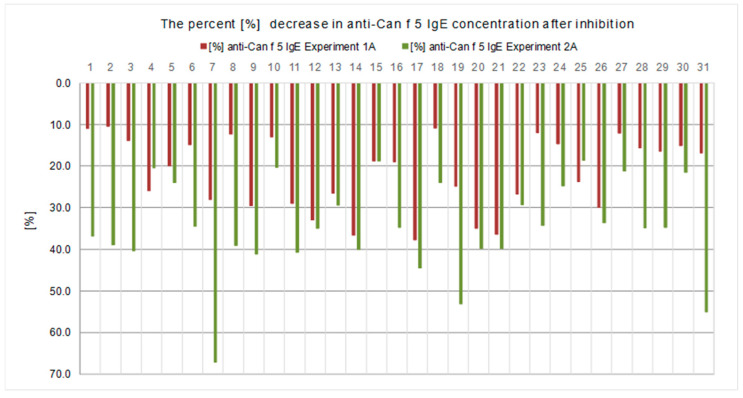
Percentage reduction in anti-Can f 5 IgE concentration, from baseline, obtained in the inhibition tests with human PSA, in each case in both experimental models used (*p* < 0.05).

**Figure 3:**
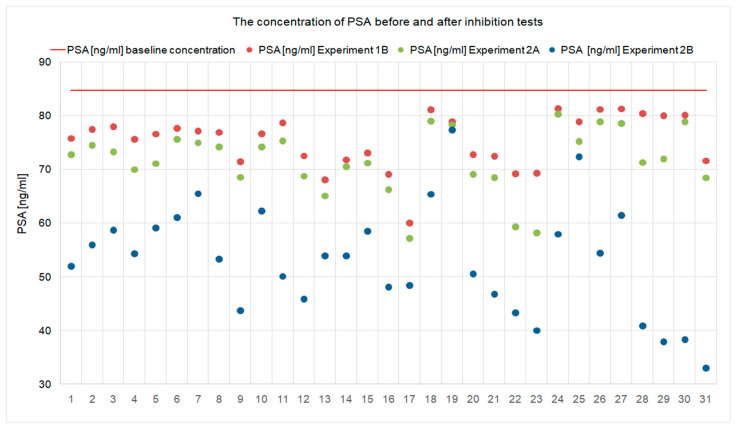
PSA concentration before and after the inhibition test with human PSA, in all experimental models used, in each individual case. The inhibition was statistically significant in each of the proposed procedures (*p* < 0.05).

**Figure 4:**
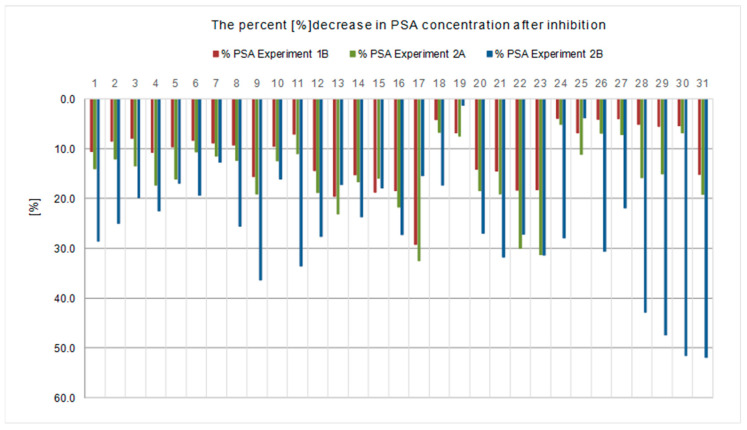
Percentage reduction of PSA concentration obtained in inhibition tests, in each individual case, in the experimental models used (*p* < 0.05).

**Figure 5:**
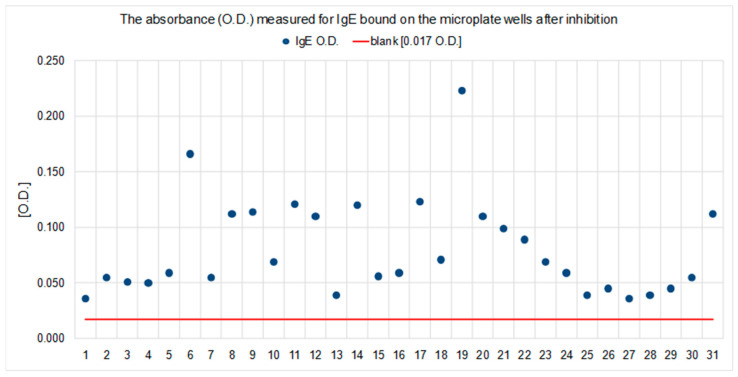
Total IgE absorbance (O.D.) was measured after the inhibition (Experiment 1B) in each individual case and was higher than that measured for the blank.

**Figure 6:**
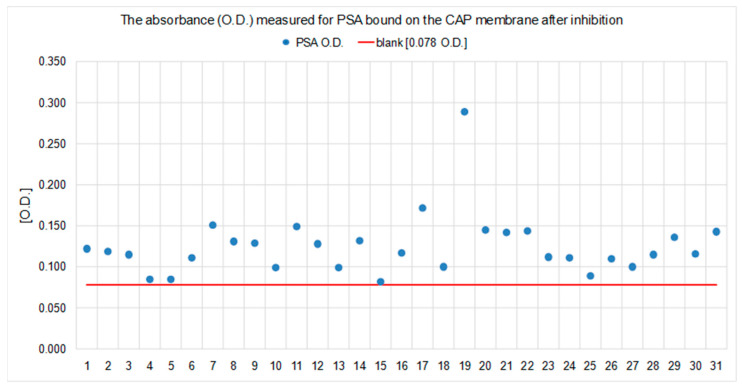
PSA absorbance (O.D.) was measured after the inhibition (Experiment 2B) in each individual case and was higher than that measured for the blank.

All results of the experiments performed in both experimental models are presented in Table 1 and Table 2 and Figure 1, Figure 2, Figure 3, Figure 4, Figure 5 and Figure 6.

Due to the small numbers of the group and the non-parametric distribution of the analytes, the statistical analysis was performed using non-parametric tests.

In the first experimental model (Experiment 1), we used the solid-phase inhibition test. We assumed that if there was cross-reactivity between canine and human kallikrein, IgE antibodies to Can f 5 (canine kallikrein) would be bound by human PSA coated on the walls of the polystyrene plate. After performing a multistage experiment, we observed that human PSA partially binds anti-Can f 5 IgE antibodies. The concentration of anti-Can f 5 IgE in the tested sera decreased after incubation in a polystyrene plate coated with human PSA (Table 1 and Table 2, Figure 1; Experiment 1A, step 4A). The decrease in concentration ranged from 10.44% to 37.73% and was observed for each test sample (Figure 2). The difference between the initial and final anti-Can f 5 concentrations was statistically significant.

The effectiveness of this blocking model was confirmed by the fact that PSA measured by ELISA, PSA specific test, in the test plate used in the inhibition (Experiment 1B, step 3B) was lower than in the calibration material used (Table 1 and Table 2, and Figure 3). The decrease in PSA concentration from the baseline value ranged from 3.98% to 29.24%, was present in each sample, and was statistically significant (Figure 4).

The presence of IgE was confirmed in the polystyrene plate used for blocking PSA-human/anti-Can f 5 (Experiment 1A, step 5A). The optical density measured after performing an ELISA reaction using HRP-labeled anti-human IgE antibodies and PNPP as a substrate was higher with respect to the blank as the first negative control (test wells with anti-Can f 5 IgEnegative serum). The results were expressed as optical density (OD) units. A positive IgE result was observed in each of the test wells of the plate used during Experiment 1 (Figure 5).

The second experimental model (Experiment 2) was based on the liquid phase inhibition test. We assumed that if IgE antibodies specific for canine kallikrein (anti-Can f 5 IgE) cross-react with human kallikrein, then mixing anti-Can f 5 positive serum with material containing PSA of a known concentration in the mixture will form complexes of “anti-Can f5 IgE/PSA human”. The formation of these complexes will cause both the PSA and anti-Can f 5 IgE concentrations in the mixture measured after incubation to be lower than the starting concentrations of both analytes, taking into account the material dilution related to the assay methodology.

The analysis of the test results showed that the PSA concentration measured in the samples after the inhibition test (Experiment 2A, step 2.1) was lower than the initial PSA concentration in the calibration material used (Figure 5). The decrease in PSA level was observed in each sample, ranging from 5.18% to 32.52%, and was statistically significant (Figure 4). A decrease in the concentration of anti-Can f 5 in the test sera (Experiment 2A, step 2.2) in relation to the initial values was also observed (Figure 1). The decrease in anti-Can f 5 levels in the test sera ranged from 18.64% to 67.09% (Figure 2) and was statistically significant.

In branch B of this experimental model (Experiment 2B), we assumed that if, after mixing anti-Can f 5 IgE positive serum with PSA-containing material, “PSA/anti-Can f 5” complexes were formed, they could be detected by binding to the membrane coated with antibodies against human immunoglobulin E. We measured the PSA concentration (Experiment 2B, step 3.1) and the anti-Can f 5 concentration (Experiment 2B, step 3.1) in the material collected after the incubation of the reaction mixture from step 1 of this experiment with a membrane coated with anti-IgE antibodies. The PSA concentration in the collected filtrates was lower than that measured in the samples after the inhibition test (Experiment 2A, step 2.1; Figure 3). The decrease in concentration ranged from 1.3% to 51.91% (Figure 4) and was statistically significant. We did not detect the presence of anti-Can f 5 IgE antibodies in the filtrates collected after the incubation of the reaction mixture from step 1 of this experiment with the membrane coated with anti-IgE antibodies (Table 1, Table 2).

Using HRP-labeled anti-PSA human antibodies and tetramethylbenzidine as chromogen, the presence of PSA (from PSA/anti-Can f 5 complexes) bound on the anti-human IgE coated membrane (Experiment 2B, step 4) was detected. The results were expressed as optical density (OD) units. The blank, which was the second negative control, was performed with a PSA calibrator and anti-Can f 5 IgE negative serum. The optical density of the filtrate after the reaction was higher than in the blank for all tested samples (Figure 6).

**Figure 7:**
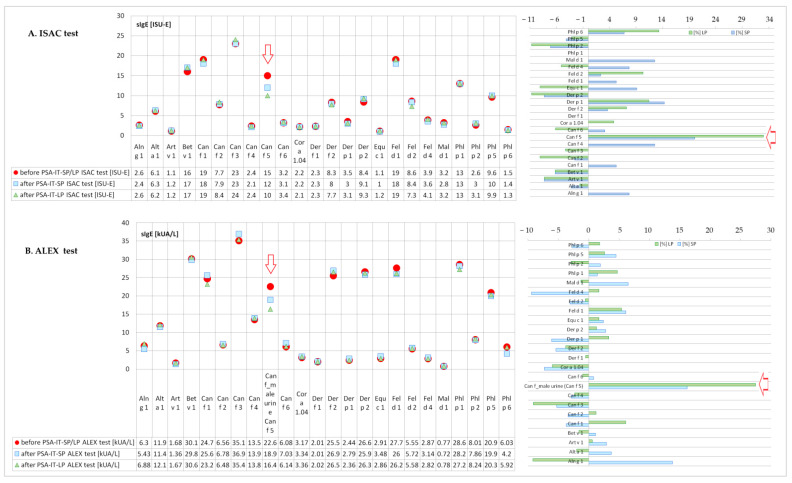
The concentration of sIgE in pooled sera ((**A**) ISAC test; (**B**) ALEX test) before and after the solid phase inhibition tests (SP-IT; Experiment 1) and in the liquid phase (LP; Experiment 2) and the percentage and direction of change. The red arrows show anti-Can f 5 IgE changes.

Evaluation of both experimental models regarding the effect specific for the IgE anti-Can f 5/PSA (human) relationship, carried out by measuring the concentration or level of specific IgE with multiplex tests (ISAC and ALEX), showed statistically insignificant, multidirectional and non-reproducible changes in the concentration of IgE-specific for allergens other than Can f 5 in any experimental model. The changes in the level of anti-Can f 5 specific IgE antibodies in the ISAC test and IgE levels specific for the canine male urine extract (Can f_male urine), which is the equivalent of Can f 5 in the ALEX test, observed after the inhibition tests (SP-IT and LP-IT) were unidirectional and reproducible in both tests and both methods of inhibition (Figure 7).

By comparing the two proposed experimental inhibition assay models (Experiment 1 and Experiment 2), we observed that both the use of the solid phase inhibition test and the liquid phase inhibition test were effective in demonstrating human kallikrein binding by canine kallikrein specific IgE antibodies. In the liquid phase inhibition test, a higher percentage decrease in the concentration of both PSA (Figure 4) and anti-Can f 5 (Figure 2) was observed from baseline than in the solid phase inhibition test. These differences were statistically significant.

## 3. Discussion

The prediction of allergen cross-reactivity is now largely based on linear sequence data or homology information between epitopes exposed on the allergen surface. Determining structure and homology is sometimes insufficient to predict the true cross-reactivity between allergens. Tests based on direct inhibitory responses seem to be useful to evaluate the clinical response associated with cross-reactivity. Various experimental models that can be used to study this phenomenon are described. However, none of them are standardized. In our study, we proposed two different experimental models with which it is possible to study cross-reactivity between different allergens. This strategy also made it possible to check whether these models give comparable results and to evaluate their effectiveness [19].

The suspicion of cross-reactivity between the human PSA and the Can f 5 canine allergen was postulated earlier [14,15]. In our previous publication, we presented the results of an inhibition test in assessing the cross-reactivity between anti-Can f 5 IgE antibodies and human PSA [17]. The protocols currently presented are an extensive, full version of that experience. The development of the inhibition test used and carrying it out with two alternative techniques allowed us to assess the usefulness of this tool in the study of cross-reactivity of allergens. It also made it possible to check whether both methods are equally effective.

Inhibition tests are a useful tool for identifying primary allergens and cross-reacting allergens. Standardized protocols for conducting this type of research are not available. The most frequently described methods are inhibition reactions based on the ELISA or ImmunoCAP technique. These tests are successfully used to test both plant and animal allergens [19,20,21]. We have already successfully used inhibition tests based on both ELISA and the ImmunoCAP technique to determine the cross-reactivity of mango, mugwort, and sunflower allergens [22,23].

Our research was based on two different test strategies to block allergen-specific antibodies with a protein with cross-reactivity potential. We used a model with the test allergen bound in the solid phase on the walls of the test wells of the ELISA plate and the model using this allergen left in the liquid phase. In both test systems, we achieved effective binding of the anti-Can f 5 IgE antibody to the human prostate antigen. Such a relationship, cross-reactivity between Can f 5 and human PSA, was previously described by Weidinger et al. [14] and Kofler et al. [15]. It was also investigated by us during an experiment that is part of the experiment described in this paper [17]. In both experimental models used, we observed a significantly lower concentration of anti-Can f 5 IgE antibodies after the human PSA inhibition reaction. This result, therefore, seems to confirm the cross-reactivity between canine kallikrein and human kallikrein and the effectiveness of the test systems used to study the cross-reactivity phenomenon. The percent blocking of anti-Can f 5 antibodies by human PSA averaged 21.60% for the solid phase inhibition assay and 34.51% for the liquid phase inhibition assay. In both experimental models, a reduction in PSA concentration was also observed after the inhibition test, compared to the baseline value. The PSA concentration decreased by an average of 11.25% in the solid phase inhibition test and by 15.49% in the liquid phase inhibition test. This observation seems to additionally confirm the effectiveness of the method used to assess the cross-reactivity of allergens. In systems where the test is carried out with the use of antibodies synthesized after natural contact with the antigen, which is found in the blood serum, obtaining 100% inhibition is unlikely. Because most antigens present many epitopes that are recognized by a large number of lymphocytes. Each lymphocyte is activated to proliferate and differentiate into plasma cells, and the resulting antibody response is polyclonal. Polyclonal antibodies are characterized by recognizing multiple epitopes on any antigen. Serum always contains a heterogeneous mixture of antibodies with different affinities. In such a case, it is always a mixture of antibodies directed against different epitopes of the same antigen in proportions depending on the efficiency of the immune system of the individual who produced them and the characteristics of the target antigen [2,24,25,26].

On the other hand, the efficiency of the inhibition process in the experimental models proposed by us may also be determined by the structure of the PSA antigen contained in the calibration material used as a source of prostate antigen. The PSA in the experiment was derived from ROCHE calibration material (ROCHE total PSA CalSet II; ref. 04485220 190). ROCHE total PSA CalSet II is a lyophilized human serum with added human PSA. Fung et al. [27] identified more than 100 proteins in the normal human seminal fluid by mass spectrometry, including the prostate serine protease (PSA) antigen and its major substrates, semenogelins I and II. Many of these proteins, including PSA, were present in multiple modified forms as a result of their post-translational modification. Protein post-translational modifications (PTMs) increase the functional diversity of the proteome by the covalent addition of functional groups or proteins, proteolytic cleavage of regulatory subunits, or degradation of entire proteins. Post-translational modifications of proteins may result in the formation of neo-epitopes which may influence the antigenicity of these proteins [28]. This, in turn, may translate into the ability of these antigens to bind both specific and cross-reactive antibodies. In terms of our experience, it may have an impact on the PSA/anti-Can f 5 IgE blocking efficacy.

Since two different blocking models were used in our experiment, it was possible to evaluate their effectiveness in the cross-reactivity study. Blocking of human PSA by anti-Can f 5 IgE was observed in both experimental models, but the effectiveness of this process was significantly higher for the liquid phase inhibition test compared to the results of the solid phase inhibition test (solid phase model vs. liquid phase model: PSA: 11.25% vs. 15.49%; anti-Can f 5 IgE: 21.60% vs. 34.51%). This result is in line with our expectations. This result is mainly influenced by the mutual availability of epitopes of the antigen and specific for my paratopes on antibody molecules. Both the liquid and solid phase inhibition methods have previously been described as useful methods for testing the cross-reactivity of various types of antigens [22,23,29,30,31,32]. In principle, the methods in the liquid phase consist in mixing proportional volumes of the antibody solution (in our case, it was anti-Can f 5 IgE positive serum) with the antigen solution (here, we used the calibration material containing human PSA). In solid-phase methods, one of the components of the antigen or antibody reaction is bound to a lower type of matrix (for example, the walls of a test tube or microplates, or cellulose). The mutual accessibility of antigens or antibodies is much greater in the case of containing them in the liquid phase than in the case of immobilization of either of them in the solid phase. Binding the antigen to solid support means that some epitopes are not available for further reactions. They are either blocked with the antibodies that bind to the antigen to the substrate, or access to them is mechanically difficult due to the proximity of the template. It, therefore, seems that the use of liquid-phase blocking should be a more effective method of testing allergen cross-reactivity than solid-phase blocking. This seems to be confirmed by the results of our research. Unfortunately, the lack of available adequate research from other centers makes it impossible to verify this hypothesis.

In the case of the experimental solid-phase inhibition model, the binding efficacy of PSA anti-Can f IgE was confirmed by detecting the presence of IgE on the walls of the polystyrene microplate after performing an inhibition test. For the liquid phase inhibition model, we expected that when mixing anti-Can f 5 IgE positive sera with PSA-containing calibration material, “anti-Can f 5 IgE/PSA human” immune complexes would form. After incubating the mixture with the ImmunCAP membrane coated with anti-human IgE antibodies, we checked the presence of PSA on this template by performing an ELISA reaction using HRP-labeled anti-PSA antibodies and TMB as the chromogen. The optical density for the test samples was higher than the optical density for the blank, which seems to confirm the binding of PSA on the ImmunCAP membrane coated with anti-IgE antibodies. This situation is only possible if PSA has been bound to anti-Can f 5 IgE during the earlier stages of the experiment. ImmunoCAP matrix factory coated with allergens have been successfully used earlier in inhibition tests to test cross-reactivity by our team [23] and other researchers [20,33,34]. However, there are no descriptions in the literature of the use of anti-IgE coated ImmunoCAP membrane to capture immune complexes composed of human IgE and any antigen. This technique appears to be a promising tool to confirm the effectiveness of removing complexes formed during liquid phase blocking tests by binding them to the ImmunoCAP matrix. The effectiveness of this technique in our experiment seems to be confirmed by the lack of anti-Can f 5 IgE antibodies in the filtrates obtained after incubation of the reaction mixture (anti-Can f 5 positive test serum/PSA calibrator).

In our opinion, both the solid phase inhibition test method and the liquid phase inhibition test method appear to be useful for assessing the actual cross-reactivity of allergens. However, the results of the experiments conducted lead us to conclude that the method of the inhibition test in the liquid phase seems to be more effective, at least in the case of using non-standardized antigens for the tests.

## 4. Materials and Methods

### 4.1. Aim of the Study and Research Group

The aim of the study was to evaluate the usefulness of various models of inhibition tests in studies on cross-reactivity of allergens. We proposed two independent research models based on the inhibition assays we described before. The ability of anti-Can f 5 IgE antibodies to block human PSA had been the subject of our research previously [17].

In the course of the experiment, we assessed the effectiveness and usefulness of two different, independent methods for studying the phenomenon of cross-reactivity between allergens on the example of the cross-reactivity assessment of canine kallikrein and human kallikrein.

In both proposed experimental models, we used sera from 31 women allergic to dogs, whose serum concentration of anti-Can f 5 IgE antibodies was at least 0.35 kU/L (≥0.35 kU/L), in accordance with common practice in the field.

### 4.2. An Experimental Model of Inhibition Testing

#### 4.2.1. Baseline Laboratory Procedure

At the beginning of the experiment, we measured the initial concentration of anti-Can f 5 IgE in all tested sera and the human-derived calibration material used (ROCHE total PSA CalSet II; ref. No. 04485220 190), which we used in the experiment.

The serum concentration of anti-Can f 5 IgE was determined by the fluoro-immuno-enzymatic method (FEIA) on ImmunCAP 100 system, using compatible reagents (Thermo Fisher Scientific, Waltham, MA, USA). ImmunoCAP is an in the vitro test system for the quantitative measurement of specific IgE in human serum or plasma. The allergen of interest, covalently coupled to ImmunoCAP, reacts with the specific IgE from the patient sample. After washing non-specific particles enzyme-labeled anti-IgE (mouse monoclonal β-galactosidase-anti-IgE) are added to form the complex. Following incubation, unbound enzyme-anti-IgE is washed away and the bound complex is then incubated with a developing agent (4-methylumbelliferyl-β-D-galactoside). After stopping the reaction (by sodium carbonate 4%), the fluorescence of the eluate is measured. The higher the response value, the more specific IgE is present in the sample. To evaluate the test results, the responses for the patient samples are transformed to concentration with the use of a calibration curve, with a calibrator range of 0–100 kUA/L.

The overall limit of quantitation for allergen-specific IgE antibodies is 0.1 kUA/L. The cross-reactivity with other human immunoglobulins is not detectable at physiological concentrations of IgA, IgM, and IgG.

The standard in vitro diagnostic (CE IVD) certified multiplex tests ImmunoCAP ISAC (Thermo Fisher Scientific, Waltham, MA, USA) and ALEX (Allergy Explorer, MacroArray Diagnostics; MADx, Vienna, Austria) were also used in the experiment.

#### 4.2.2. Experiment Model 1 (Experiment 1)—The Solid Phase Inhibition Model (SP-IT)

The first experimental model was based on a polystyrene microplate ELISA test (Total Prostate-Specific Antigen ELISA (ref. 25-PSAHU-E01), ALPCO Salem, NH, USA) coated with goat anti-human PSA antibody, and after the first two stages, it was carried out in two independent branches.

As a source of human PSA with a known concentration in our experiment, we used the ROCHE calibration solution (ROCHE total PSA CalSet II; ref. 04485220 190; Roche Diagnostics GmbH, Mannheim, Germany). Total PSA CalSet II is a lyophilized human serum with added human PSA. The PSA concentration declared by the manufacturer in this material is 58.5 ng/mL. The concentration of PSA in this calibrator as measured by the ALPCO assay that was used in the experiment was 84.75 ng/mL. We took this value into account in later calculations.

Before starting the experiment, we checked that the serum from the women participating in the study did not contain a detectable concentration of PSA. The calibrator used was free of anti-Can f 5 IgE. There were also no IgE antibodies specific for any of the allergens represented in the ISAC and ALEX tests.

**Figure 8:**
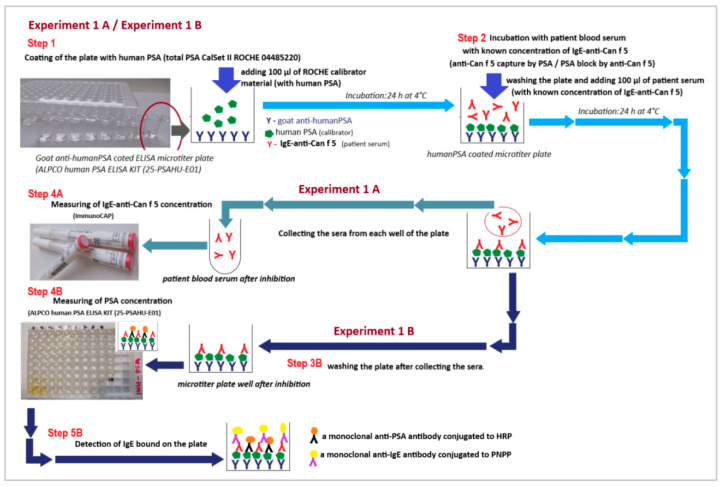
Scheme of the solid-phase inhibition test (SP-IT)—experimental model 1 (Experiment 1).

A schematic diagram of this experiment model (Experiment 1) is shown in Figure 8.

The results of this experiment model (Experiment 1) are shown in Table 1 and Table 2 and Figure 1, Figure 2, Figure 3, Figure 4 and Figure 5.

*Experiment 1 (branch A and branch B)*
*Step 1:*

In the first step of this experiment, the plate was coated with human PSA. For this purpose, 100 µL of the ROCHE calibration solution containing human PSA was added to all microplate wells. The plate, prepared in this way, was then incubated at 4 °C for 24 h to immobilize PSA from the calibrator on the walls of the test wells of the plate. After this time, the calibrator was removed and wells were washed 3 times with distilled water to remove any unbound residues and clean wells. This procedure produced microplate wells coated with human PSA. This microplate was then used, in the second step of this experiment, to bound Can f 5 specific IgE (anti-Can f 5 IgE) from patients’ serum with human PSA coated to the walls of microwells of the polystyrene plate.

*Step 2:*

One hundred microliters of the patient serum (with known anti-Can f 5 IgE concentration) was added (in duplicate) into the appropriate wells of the polystyrene plate, pre-coated with human PSA. The prepared plate was incubated at 4 °C for 24 h. We assumed that during this step, anti-Can f 5 IgE from patients’ serum would bind to the PSA coated on the microplate wells.

*Step 3:*

After incubation (step 3A), patients sera were collected from microwells to the clean polystyrene tubes and after collecting sera, the plate was washed 5 times with distilled water (step 3B). Collected sera were used in branch A (Experiment 1A) and microplate wells were used in branch B (Experiment 1B) in this experimental model.

*Experiment 1A*
*Step 4A:*

In this branch of the experiment, we assumed that some of the anti-Can f 5 (IgE) antibodies from the test sera were bound by PSA immobilized on walls of microplate wells. We expected to reduce the concentration of anti-Can f 5 (IgE) in the test sera relative to the baseline value. To verify this assumption, during this step, the concentration of anti-Can f 5 IgE was determined in each serum collected from microplate wells at the end of Step 2(ImmunoCAP;). The results for this branch of the experiment are shown in Table 1 and Table 2, Figure 1 and Figure 2.

*Experiment 1B*
*Step 4B:*

In this branch of the experiment, we assumed that a portion of the PSA bound to the walls of the microplate during the first stage (Step 1) of the experiment was blocked by anti-Can f 5 IgE. We expected that the PSA concentration measured at this stage would be lower than that measured baseline in the ROCHE calibrator used (84.75 ng/mL). To verify this assumption, the concentration of PSA bound in each test well of the plate was determined according to the procedure provided by the manufacturer of the test (ALPCO; Total Prostate-Specific Antigen ELISA (ref. 25-PSAHU-E01)). One hundred microliters of Enzyme Conjugate Reagent (monoclonal anti-PSA-HRP(horseradish peroxidase) conjugate) was dispensed to the washed plate into each well. The plate was incubated (18–25 °C; 60 min) and then washed again (deionized water) 5 times, and 100 µL of a TMB Reagent (tetramethyl benzidine) was dispensed into each well. After 20 min of incubation (18–25 °C)) in dark, the reaction was stopped by adding 100 µL of the Stop Solution (1N HCl) to each well. Then, the optical density (450 nm) was read and the PSA concentration was calculated based on the simultaneously prepared calibration curve (0, 2, 4, 15, 60, and 120 ng/mL PSA).

The results for this branch of the experiment are shown in Table 1 and Table 2, Figure 3 and Figure 4.

*Step 5B:*

In this step of the experiment, we assumed that if the anti-Can f 5 of the test sera was bound by PSA on the walls of the microplate, it was possible to detect total IgE in that plate. To test the validity of this hypothesis, we checked the presence of IgE in the test plate used in the experiment. To detect the presence of IgE on the walls of the microplate, we used reagents dedicated to the determination of human IgE in serum, which is part of the validated HYCOR kit (HYTEC Specific and Total IgE EIA kit). The kit uses mouse anti-human IgE antibodies conjugated with alkaline phosphatase and a dedicated substrate p-nitro-phenyl-phosphate (PNPP). We have manually followed all steps of the manufacturer’s recommended procedure for selected elements of the procedure.

After step 4B, the plate was rinsed 3 times with a washing solution dedicated to the method used (saline solution with Triton X-100). An appropriate volume of enzyme-conjugated mouse anti-human IgE antibodies was then added to each test well. After incubation (37 °C; 30 min), the plate was washed again (as before) and substrate was added. After incubation with the substrate (1 h; 37 °C), the reaction was stopped (1N sodium hydroxide solution) and the absorbance was measured for each test well (450 nm/630 nm). Due to the lack of possibility of calibration, the concentration of IgE was not calculated. The results were considered positive if the measured absorbance (O.D.) was higher than the blank (for A1, and B1 wells microplates that were not coated with PSA throughout the experiment). The results for this branch of the experiment are shown in Table 1 and Table 2 and Figure 5.

#### 4.2.3. Experiment Model 2 (Experiment 2)—The Liquid Phase Inhibition Model (LP-IT)

The second experimental model was based on the blocking of PSA-anti-Can f 5 (IgE) as a result of mixing patient sera with the PSA calibrator solution. The same calibration material (ROCHE total PSA CalSet II; ref. 04485220 190) was used as in the previous experimental model.

**Figure 9:**
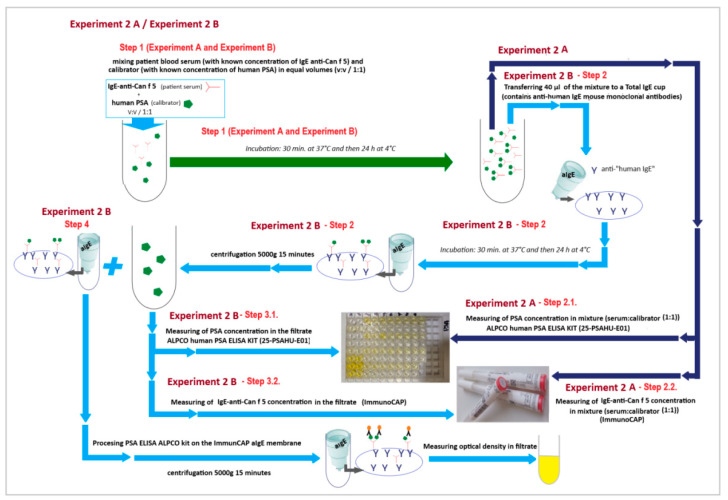
Scheme of the liquid phase inhibition test (LP-IT)—experimental model 2 (Experiment 2).

A schematic diagram of this experiment model (Experiment 2) is shown in Figure 9.

The results of this experiment model (Experiment 2) are shown in Table 1 and Table 2, Figure 1, Figure 2, Figure 3 and Figure 4 and Figure 6.

*Experiment 2 (branch A and branch B)*
*Step 1—Experiment 2A and Experiment 2B:*

The first stage was common to all branches (2A and 2B) of this experimental model. The experiment started with mixing the sera of patients with a calibrator with a known concentration of PSA (84.75 ng/mL) in equal volumes (V:V; 1:1). The samples prepared in this way were then subjected to a two-stage incubation: first at 37 °C for 30 min and then 24 h at 4 °C. After the incubation was completed, the material was divided into two branches of the experiment (Experiment 2A and Experiment 2B).

*Experiment 2A*
*Step 2A:*

In this branch of the experiment, we assumed that serum anti-Can f 5 IgE would be bound to PSA derived from the calibration material. We expected a decrease in the concentration of both anti-Can f 5 and PSA about the baseline values of these parameters. To verify the validity of the assumed hypothesis, the concentrations of anti-Can f 5 IgE (ImmunoCAP) and PSA (ALPCO; Total Prostate-Specific Antigen ELISA (ref. 25-PSAHU-E01)) were measured in the sample after incubation. Both procedures were described previously.

The material dilution was included in all calculations. At the beginning of the experiment, we also checked that the serum from the women participating in the study did not contain detectable concentrations of PSA (ALPCO procedure) and that the calibrator used was free of anti-Can f 5 IgE (ImmunoCAP procedure).

*Experiment 2B*
*Step 2 (Experiment 2B)*

During this stage of the experiment, we assumed that if the “PSA-anti-Can f 5 IgE” complexes were formed in the first step of the experiment, it is possible to remove them from the mixture using antibodies specific for human immunoglobulin E. To check the soundness of our assumption, the mixture from the first stage of the experiment was applied to cellulose supports coated with mouse anti-human IgE monoclonal antibodies.

ImmunoCAP reaction cups for the determination of total serum IgE concentration (ImmunoCAP; 14-4509-01) were used in the experiment. These cups are part of the system used to determine the concentration of total IgE in human serum in the range of 2.0 to 5000 kU/L. During the experiment, 40 µL of each mixture from step 1 was applied to a reaction cup coated with anti-IgE antibodies. Before this, all cups were pre-rinsed with a dedicated washing buffer (ImmunoCAP; 10-9422-01) and dried (centrifugation 5000× *g*, 15 min). We decided to use that mixture volume (40 µL) because it is the standard volume of material to which the ImmunoCAP reaction cups are calibrated (ImmunoCAP Total IgE Direction for Use 52-5292-EN/04). The reaction cups with the mixture from the experiment in Step 1 applied in this way were closed in Eppendorf tubes (which prevented evaporation) and were incubated for 30 min at 37 °C and then 24 h at 4 °C. After incubation, the reaction cups sealed in the tubes were centrifuged (5000× *g*; 15 min) to collect the filtrate.

*Step 3.1. (Experiment 2B)*

We measured the concentration of PSA (ALPCO; Total Prostate-Specific Antigen ELISA (ref. 25-PSAHU-E01)) in the collected filtrate. We expected that if “PSA-anti-Can f 5 IgE” immune complexes were formed in Step 1 of Experiment 2 (A/B) and they were bound by anti-IgE antibodies in Step 2 of Experiment 2B, the PSA concentration in the filtrate was below the value that we measured during Step 2.1. of Experiment 2A and the baseline PSA concentration were determined in the calibration material before all experiments.

*Step 3.2. (Experiment 2B)*

We measured the concentration of anti-Can f 5 IgE in the collected filtrates (ImmunoCAP). We expected that if “PSA-anti-Can f 5 IgE” immune complexes were formed in Step 1 of Experiment 2 and they were bound by anti-IgE antibodies in Step 2 of Experiment 2B, the anti-Can f 5 IgE concentration in the filtrate was below the value that we measured during Step 2.2. of Experiment 2A and the baseline anti-Can f 5 IgE concentration was measured in the sera at the beginning of the experiments.

During all analyzes and calculations, we took into account the occurring material dilutions.

*Step 4 (Experiment 2B)*

In this part of the experiment, we assumed that if “anti-Can f 5 IgE/PSA” complexes were bound to the ImmunoCAP membrane coated with human anti-IgE antibodies, it was possible to detect PSA from these complexes on the membrane surface. To check whether our assumptions are true, in order to detect bound PSA, we performed an ELISA test using anti-PSA human antibodies labeled with HRP and TMB as a chromogen. For this, we 100 µL of the monoclonal anti-PSA-HRP conjugate was dispensed to the washed reaction cups. All cups were incubated (18–25 °C; 60 min). After incubation, each cup was washed with deionized water 5 times. For this purpose, 100 mL of water were poured into each cup and centrifuged (15 min, 5000× *g*). The filtrate was discarded. To rinse the cups, 100 µL of TMB was added to each. After 20 min of incubation (18–25 °C) in dark, the reaction was stopped by adding 100 µL of Stop Solution (1N HCl) to each well. After stopping the enzymatic reaction, all cups were centrifuged (15 min., 5000× *g*) and the optical density (450 nm) was measured in the collected filtrate. A blank test was run simultaneously with a clean algE reaction cup. The optical density measured in the filters was compared with the blank value. We assumed that if PSA was bound on the aIgE-CAP membrane, the optical density of the test samples would be higher than that of the blank.

### 4.3. Controls

In the experiment, we used two negative controls, adequate to the individual stages of the experiment.

In experimental model 1 (solid phase inhibition test; SP-IT), as a negative control (first negative control), we used sera in which no anti-Can f 5 IgE antibodies were detected (anti-Can f 5 IgE concentration were lower than 0.1 kUA/L). In experimental model 2 (liquid phase inhibition test; LP-IT), as a negative control (second negative control), we used a mixture of sera in which no anti-Can f 5 IgE antibodies were detected (anti-Can f 5 IgE concentration were lower than 0.1 kUA/L) and the calibration material (ROCHE total PSA CalSet II; ref. No. 04485220 190) that was the source of the human PSA used in the experiment. The same procedures were applied to both controls, which were used for all test samples, adequately to the particular model of experiment to which the control was referring.

In order to control and evaluate the performed experiments and to exclude the unexpected blocking of IgE antibodies specific for non-Can f 5 alergens by PSA, the concentration or level of specific IgE was measured using two independent, standard, multiparameter platforms certified for in vitro diagnostics (CE IVD): the ALEX test (Allergy Explorer, MacroArray Diagnostics; MADx, Vienna, Austria) and ImmunoCAP ISAC (Thermo Fisher Scientific, Waltham, MA, USA) in pooled sera. The tests were performed in three portions of pooled sera and in calibration material, which was the source of PSA (ROCHE total PSA CalSetII; ref. No. 04485220190). Sera were pooled at three experimental points: before the experiments, after incubation on the PSA coated plate (Experiment 1), and after incubation of the serum mixed with the PSA calibrator in a 1:1 volume ratio (Experiment 2). The sIgE results positive for allergens, which are presented in both tests (ISAC and ALEX), were used for the analysis. Material dilution was taken into account where necessary in the evaluation of the results.

### 4.4. Limitations and Future Perspectives

We are aware that our experimental models have some limitations related to the research tools used and the assumed inclusion criteria.

The study group did not include men allergic to Can f 5. It cannot be ruled out that restricting the group to individuals of one sex may affect the results of the study. In our opinion, however, in the case of men, it would be necessary to take into account the effect of PSA, which is naturally present in male serum. This would certainly require the design of a different experimental model and seems to be an interesting direction for the development of the presented research.

It would probably also be advisable to extend the presented experiment to anti-Can f 5 IgE inhibition assays with proteins other than PSA. Such a strategy would require the definition of unambiguous criteria for the selection of proteins, which are known for certain not to cross-react with the analytes tested in the research system used by us. It certainly requires further, more extensive research.

We wanted to present simple models for studying the phenomenon of cross-reactivity between allergens. The presented experimental models certainly have their limitations and require further research. It seems, however, that in the absence of standardized methods to study this phenomenon, each research proposal may increase knowledge in this field.

### 4.5. Statistical Analysis

Statistical analysis: Shapiro-Wilk Test, Wilcoxon Signed Rank Test, and ANOVA Friedman Test were used. Analyses were prepared using the Statistica Software, version 13.3, and Microsoft Excel Software.

## 5. Conclusions

Due to the lack of standardized methods for testing cross-reactivity between allergens, inhibition tests seem to be a useful and effective experimental tool for this purpose. They can be used in both solid and liquid phases. Inhibition in the liquid phase seems to be a more effective research tool. This is probably because leaving the components of the immune reaction in suspension increases the mutual availability of antibodies and antigenic epitopes. In the solid phase reaction model, antigens are bound by antibodies immobilized on the walls of a polystyrene plate, which limits the availability of certain antigenic epitopes to the antibodies and may reduce the efficiency of the reaction.

The conducted experiments also seem to confirm the partial cross-reactivity between canine and human kallikrein, which may be of significant clinical importance in the case of seminal fluid hypersensitivity observed in some women allergic to dogs.

## Figures and Tables

**Table 1:** The results of both models inhibition tests in each individual case.

Indiwidual Case	anti-Can f 5 IgE [kUA/L] *Baseline (before Inhibition)*	anti-Can f 5 IgE [kUA/L] *after Inhibition Experiment 1A*	anti-Can f 5 IgE [kUA/L] *after Inhibition Experiment 2A*	[%] Decrease anti-Can f 5 IgE *Experiment 1A*	[%] Decrease anti-Can f 5 IgE *Experiment 2A*	PSA [ng/mL] *Experiment 1B*	PSA [ng/mL] *Experiment 2A*	PSA [ng/mL] *Experiment 2B*	[%] Decrease PSA *Experiment 1B*	[%] Decrease PSA *Experiment 2A*	[%] Decrease PSA *Experiment 2B*	IgE [O.D.] *Experiment 1B*	PSA [O.D.] *Experiment 2B*
1	4.92	4.38	3.11	11.0	36.8	75.80	72.80	51.95	10.6	14.1	28.6	0.036	0.122
2	3.83	3.43	2.34	10.4	38.9	77.50	74.50	55.88	8.6	12.1	25.0	0.055	0.119
3	2.38	2.05	1.42	13.9	40.3	77.98	73.29	58.69	8.0	13.5	19.9	0.051	0.115
4	0.54	0.40	0.43	25.9	20.4	75.64	70.01	54.28	10.8	17.4	22.5	0.050	0.085
5	0.50	0.40	0.38	20.0	24.0	76.60	71.12	59.06	9.7	16.1	17.0	0.059	0.085
6	1.48	1.26	0.97	14.9	34.5	77.66	75.66	60.97	8.4	10.7	19.4	0.166	0.111
7	23.40	16.83	7.70	28.1	67.1	77.18	74.98	65.44	8.9	11.5	12.7	0.055	0.151
8	6.76	5.93	4.12	12.3	39.1	76.92	74.22	53.23	9.2	12.4	25.6	0.112	0.131
9	5.22	3.68	3.07	29.5	41.2	71.45	68.58	43.66	15.7	19.1	36.3	0.114	0.129
10	0.69	0.60	0.55	13.0	20.3	76.65	74.21	62.26	9.6	12.4	16.1	0.069	0.099
11	20.40	14.50	12.10	28.9	40.7	78.72	75.36	50.04	7.1	11.1	33.6	0.121	0.149
12	5.13	3.44	3.34	32.9	34.9	72.51	68.78	45.79	14.4	18.8	27.6	0.110	0.128
13	1.09	0.81	0.77	26.5	29.4	68.10	65.12	53.86	19.6	23.2	17.3	0.039	0.099
14	8.85	5.79	5.31	36.6	40.0	71.82	70.59	53.86	15.3	16.7	23.7	0.120	0.132
15	0.48	0.39	0.39	18.8	18.8	73.09	71.22	58.49	18.8	16.0	17.9	0.056	0.082
16	3.00	2.43	1.96	19.0	34.7	69.11	66.31	48.02	18.5	21.8	27.3	0.059	0.117
17	34.40	21.42	19.10	37.7	44.5	59.97	57.19	48.34	29.2	32.5	15.5	0.123	0.172
18	0.92	0.82	0.70	10.9	23.9	81.17	79.01	65.34	4.2	6.8	17.3	0.071	0.100
19	75.70	56.88	35.50	24.9	53.1	78.94	78.36	77.34	6.9	7.5	1.3	0.223	0.289
20	8.69	5.65	5.23	35.0	39.8	72.77	69.11	50.46	14.1	18.5	27.0	0.110	0.145
21	7.30	6.64	4.39	36.4	39.9	72.46	68.54	46.75	14.5	19.1	31.8	0.099	0.142
22	7.06	5.18	4.99	26.7	29.3	69.16	59.36	43.24	18.4	30.0	27.2	0.089	0.144
23	1.78	1.59	1.17	12.0	34.3	69.27	58.21	39.95	18.3	31.3	31.4	0.069	0.112
24	1.09	0.93	0.82	14.7	24.8	81.38	80.36	57.90	4.0	5.2	28.0	0.059	0.111
25	0.59	0.45	0.48	23.7	18.6	78.94	75.27	72.34	6.9	11.2	3.9	0.039	0.089
26	1.07	0.75	0.71	29.9	33.6	81.22	78.90	54.39	4.2	6.9	30.7	0.045	0.110
27	0.99	0.87	0.78	12.1	21.2	81.33	78.63	61.41	4.0	7.2	21.9	0.036	0.100
28	1.15	0.97	0.75	15.7	34.8	80.42	71.33	40.80	5.1	15.8	42.8	0.039	0.115
29	2.82	2.36	1.84	16.4	34.8	80.05	71.96	37.82	5.6	15.1	47.5	0.045	0.136
30	0.93	0.79	0.73	15.1	21.5	80.16	78.94	38.25	5.4	6.9	51.6	0.055	0.116
31	19.70	16.38	8.87	16.9	55.0	71.61	68.47	32.93	15.2	19.2	51.9	0.112	0.143

Legend: O.D.—Optical density.

**Table 2:** The average results of both models of inhibition tests.

	Median	Min.	Max.	Average	SD
**anti-Can f 5 IgE baseline (before inhibition) [kUA/L]**	2.82	0.48	75.70	8.16	14.87
**anti-Can f 5 IgE after inhibition Experiment 1A [kUA/L]**	2.36	0.39	56.88	6.06	10.91
**anti-Can f 5 IgE after inhibition Experiment 2A [kUA/L]**	1.84	0.38	35.50	4.32	7.07
**anti-Can f 5 IgE after inhibition Experiment 2B [kUA/L]**	--------	<0.1	<0.1	--------	--------
**[%] anti-Can f 5 IgE Experiment 1A [kUA/L]**	19.00	10.44	37.73	21.60	8.79
**[%] anti-Can f 5 IgE Experiment 2A [kUA/L]**	34.75	18.64	67.09	34.51	11.25
**PSA baseline concentration [ng/mL]**	84.75	84.75	84.75	84.75	0.00
**PSA Experiment 1B [ng/mL]**	76.65	59.97	81.38	75.34	4.99
**PSA Experiment 2A [ng/mL]**	71.96	57.19	80.36	71.63	5.97
**PSA Experiment 2B [ng/mL]**	53.86	32.93	77.34	52.99	10.22
**[%] PSA Experiment 1B**	9.56	3.98	29.24	11.25	6.02
**[%] PSA Experiment 2A**	15.09	5.18	32.52	15.49	7.04
**[%] PSA Experiment 2B**	25.59	1.30	51.91	25.81	11.88

## Data Availability

Not applicable.
